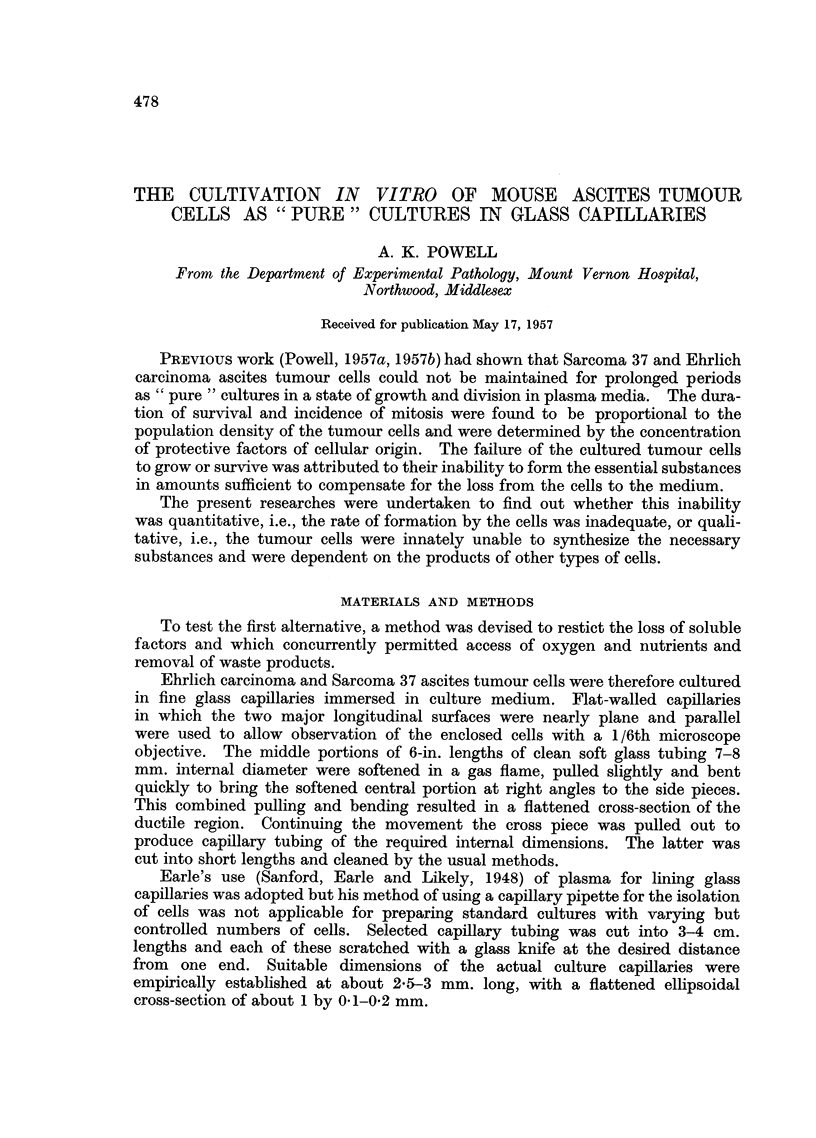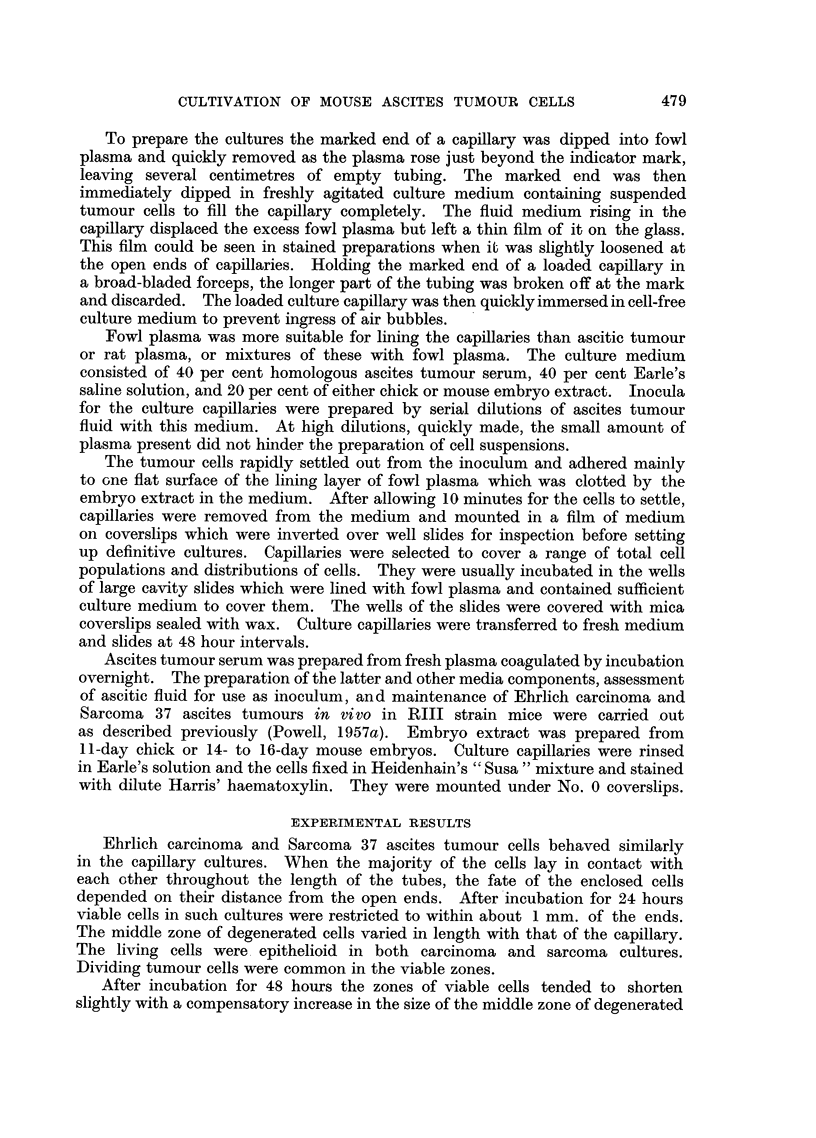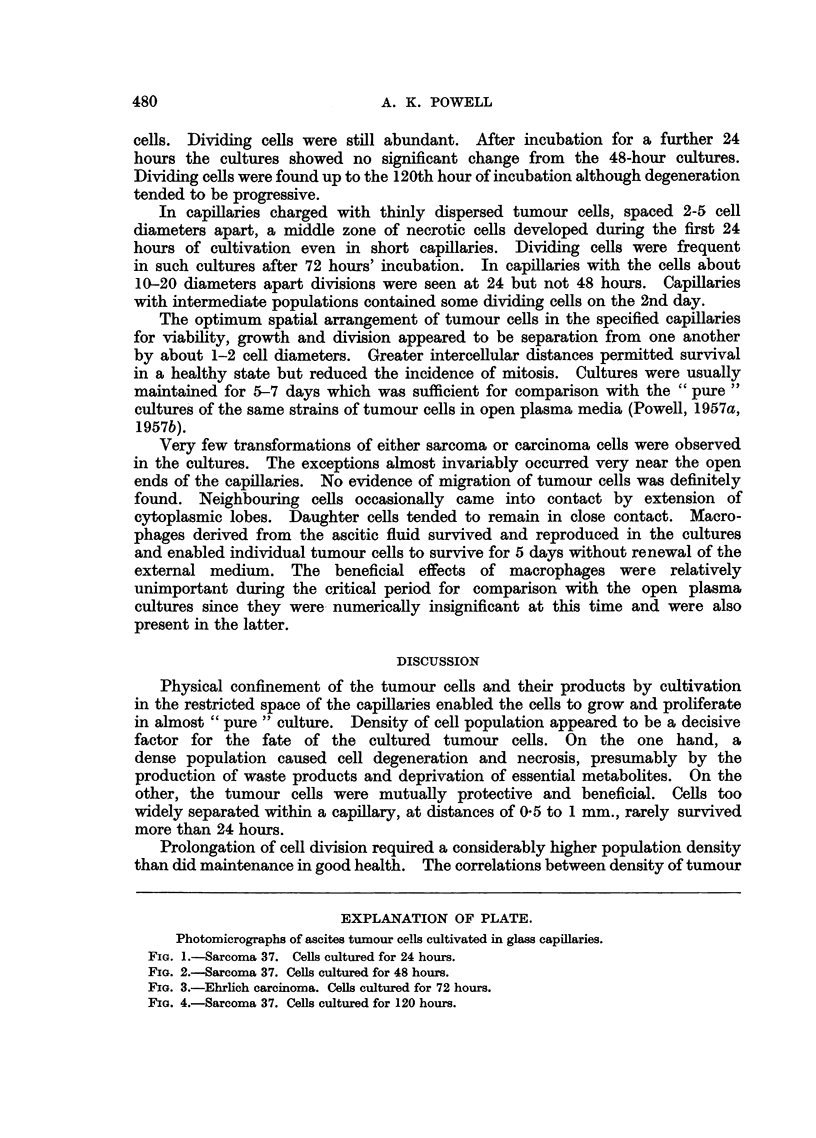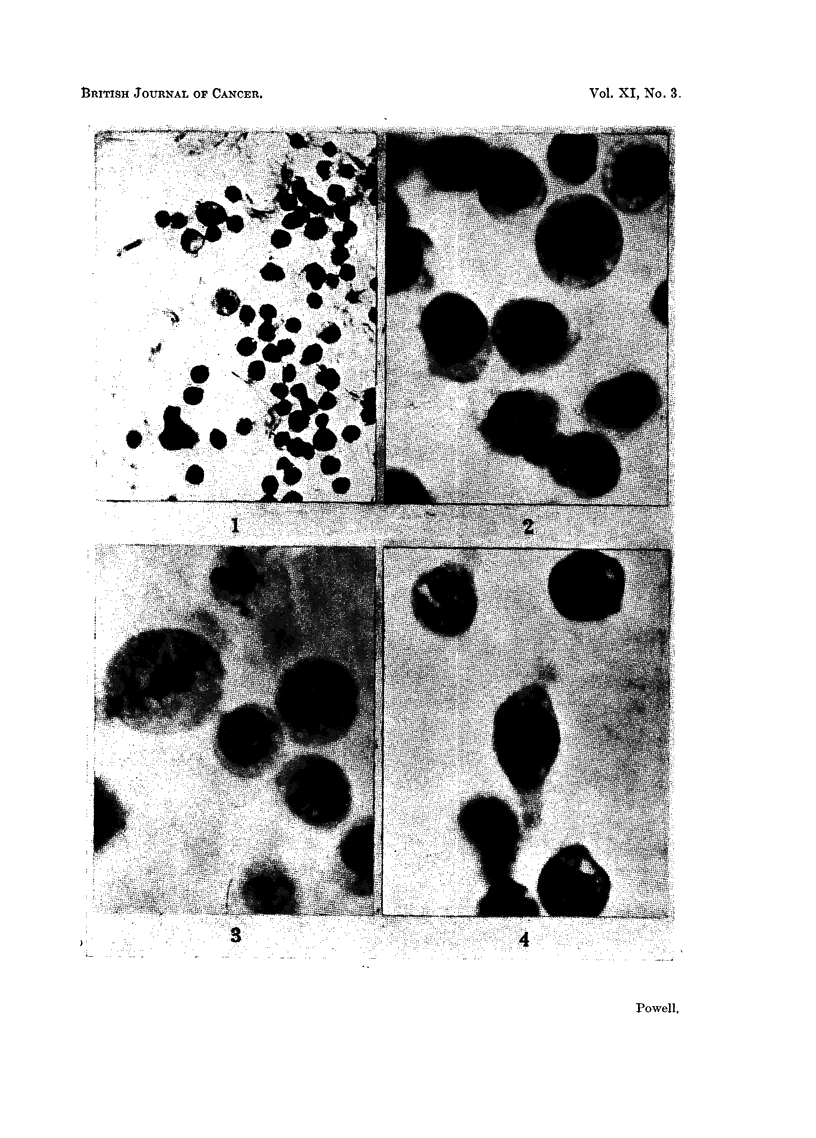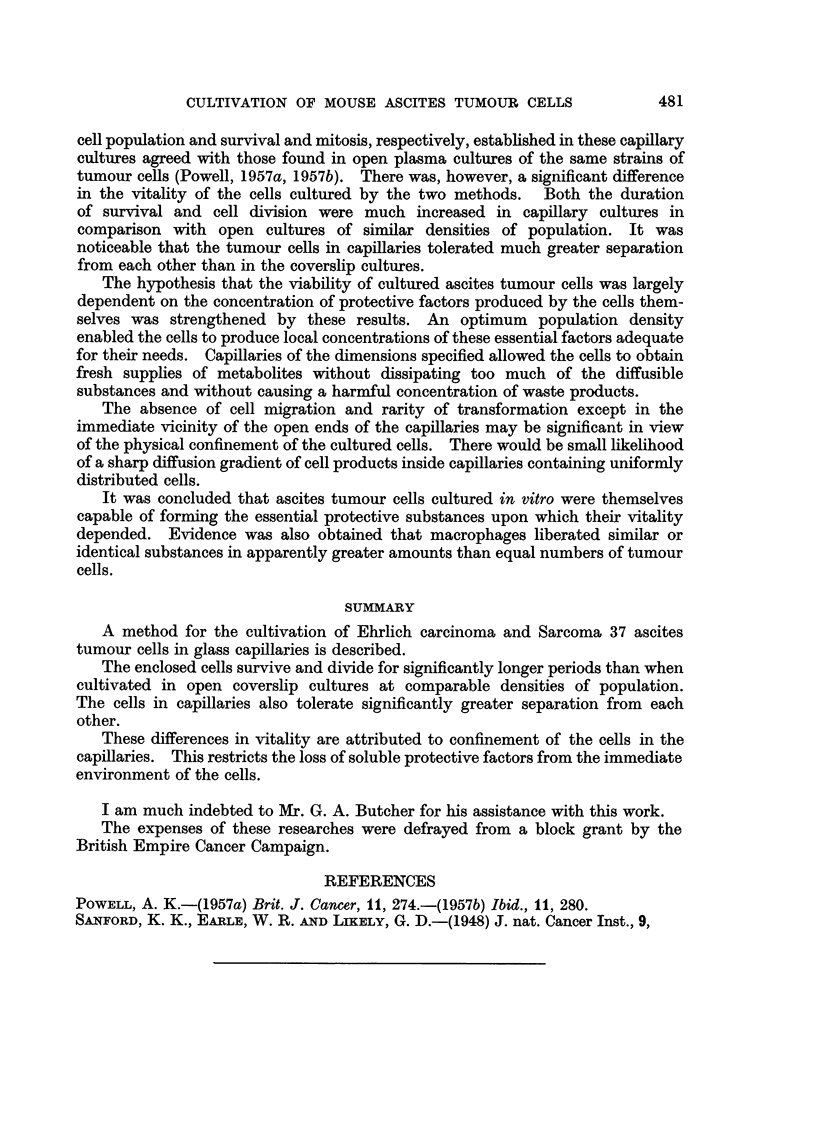# The Cultivation in vitro of Mouse Ascites Tumour Cells as “Pure” Cultures in Glass Capillaries

**DOI:** 10.1038/bjc.1957.57

**Published:** 1957-09

**Authors:** A. K. Powell

## Abstract

**Images:**


### THE CULTIVATION *IN VITRO* OF MOUSE ASCITES TUMOUR CELLS AS " PURE " CULTURES IN GLASS CAPILLARIES

A. K. POWELL

*From the Department of Experimental Pathology, Mount Vernon Hospital, Northwood, Middlesex*

Received for publication May 17, 1957

PREVIOUS work (Powell, 1957*a*, 1957*b*) had shown that Sarcoma 37 and Ehrlich carcinoma ascites tumour cells could not be maintained for prolonged periods as " pure " cultures in a state of growth and division in plasma media. The duration of survival and incidence of mitosis were found to be proportional to the population density of the tumour cells and were determined by the concentration of protective factors of cellular origin. The failure of the cultured tumour cells to grow or survive was attributed to their inability to form the essential substances in amounts sufficient to compensate for the loss from the cells to the medium.

The present researches were undertaken to find out whether this inability was quantitative, i.e., the rate of formation by the cells was inadequate, or qualitative, i.e., the tumour cells were innately unable to synthesize the necessary substances and were dependent on the products of other types of cells.

### MATERIALS AND METHODS

To test the first alternative, a method was devised to restict the loss of soluble factors and which concurrently permitted access of oxygen and nutrients and removal of waste products.

Ehrlich carcinoma and Sarcoma 37 ascites tumour cells were therefore cultured in fine glass capillaries immersed in culture medium. Flat-walled capillaries in which the two major longitudinal surfaces were nearly plane and parallel were used to allow observation of the enclosed cells with a 1/6th microscope objective. The middle portions of 6-in. lengths of clean soft glass tubing 7–8 mm. internal diameter were softened in a gas flame, pulled slightly and bent quickly to bring the softened central portion at right angles to the side pieces. This combined pulling and bending resulted in a flattened cross-section of the ductile region. Continuing the movement the cross piece was pulled out to produce capillary tubing of the required internal dimensions. The latter was cut into short lengths and cleaned by the usual methods.

Earle's use (Sanford, Earle and Likely, 1948) of plasma for lining glass capillaries was adopted but his method of using a capillary pipette for the isolation of cells was not applicable for preparing standard cultures with varying but controlled numbers of cells. Selected capillary tubing was cut into 3–4 cm. lengths and each of these scratched with a glass knife at the desired distance from one end. Suitable dimensions of the actual culture capillaries were empirically established at about 2·5–3 mm. long, with a flattened ellipsoidal cross-section of about 1 by 0·1–0·2 mm.

To prepare the cultures the marked end of a capillary was dipped into fowl plasma and quickly removed as the plasma rose just beyond the indicator mark, leaving several centimetres of empty tubing. The marked end was then immediately dipped in freshly agitated culture medium containing suspended tumour cells to fill the capillary completely. The fluid medium rising in the capillary displaced the excess fowl plasma but left a thin film of it on the glass. This film could be seen in stained preparations when it was slightly loosened at the open ends of capillaries. Holding the marked end of a loaded capillary in a broad-bladed forceps, the longer part of the tubing was broken off at the mark and discarded. The loaded culture capillary was then quickly immersed in cell-free culture medium to prevent ingress of air bubbles.

Fowl plasma was more suitable for lining the capillaries than ascitic tumour or rat plasma, or mixtures of these with fowl plasma. The culture medium consisted of 40 per cent homologous ascites tumour serum, 40 per cent Earle's saline solution, and 20 per cent of either chick or mouse embryo extract. Inocula for the culture capillaries were prepared by serial dilutions of ascites tumour fluid with this medium. At high dilutions, quickly made, the small amount of plasma present did not hinder the preparation of cell suspensions.

The tumour cells rapidly settled out from the inoculum and adhered mainly to one flat surface of the lining layer of fowl plasma which was clotted by the embryo extract in the medium. After allowing 10 minutes for the cells to settle, capillaries were removed from the medium and mounted in a film of medium on coverslips which were inverted over well slides for inspection before setting up definitive cultures. Capillaries were selected to cover a range of total cell populations and distributions of cells. They were usually incubated in the wells of large cavity slides which were lined with fowl plasma and contained sufficient culture medium to cover them. The wells of the slides were covered with mica coverslips sealed with wax. Culture capillaries were transferred to fresh medium and slides at 48 hour intervals.

Ascites tumour serum was prepared from fresh plasma coagulated by incubation overnight. The preparation of the latter and other media components, assessment of ascitic fluid for use as inoculum, and maintenance of Ehrlich carcinoma and Sarcoma 37 ascites tumours *in vivo* in RIII strain mice were carried out as described previously (Powell, 1957*a*). Embryo extract was prepared from 11-day chick or 14- to 16-day mouse embryos. Culture capillaries were rinsed in Earle's solution and the cells fixed in Heidenhain's "Susa" mixture and stained with dilute Harris' haematoxylin. They were mounted under No. 0 coverslips.

### EXPERIMENTAL RESULTS

Ehrlich carcinoma and Sarcoma 37 ascites tumour cells behaved similarly in the capillary cultures. When the majority of the cells lay in contact with each other throughout the length of the tubes, the fate of the enclosed cells depended on their distance from the open ends. After incubation for 24 hours viable cells in such cultures were restricted to within about 1 mm. of the ends. The middle zone of degenerated cells varied in length with that of the capillary. The living cells were epithelioid in both carcinoma and sarcoma cultures. Dividing tumour cells were common in the viable zones.

After incubation for 48 hours the zones of viable cells tended to shorten slightly with a compensatory increase in the size of the middle zone of degenerated cells. Dividing cells were still abundant. After incubation for a further 24 hours the cultures showed no significant change from the 48-hour cultures. Dividing cells were found up to the 120th hour of incubation although degeneration tended to be progressive.

In capillaries charged with thinly dispersed tumour cells, spaced 2-5 cell diameters apart, a middle zone of necrotic cells developed during the first 24 hours of cultivation even in short capillaries. Dividing cells were frequent in such cultures after 72 hours' incubation. In capillaries with the cells about 10–20 diameters apart divisions were seen at 24 but not 48 hours. Capillaries with intermediate populations contained some dividing cells on the 2nd day.

The optimum spatial arrangement of tumour cells in the specified capillaries for viability, growth and division appeared to be separation from one another by about 1–2 cell diameters. Greater intercellular distances permitted survival in a healthy state but reduced the incidence of mitosis. Cultures were usually maintained for 5–7 days which was sufficient for comparison with the " pure " cultures of the same strains of tumour cells in open plasma media (Powell, 1957*a*, 1957*b*).

Very few transformations of either sarcoma or carcinoma cells were observed in the cultures. The exceptions almost invariably occurred very near the open ends of the capillaries. No evidence of migration of tumour cells was definitely found. Neighbouring cells occasionally came into contact by extension of cytoplasmic lobes. Daughter cells tended to remain in close contact. Macrophages derived from the ascitic fluid survived and reproduced in the cultures and enabled individual tumour cells to survive for 5 days without renewal of the external medium. The beneficial effects of macrophages were relatively unimportant during the critical period for comparison with the open plasma cultures since they were numerically insignificant at this time and were also present in the latter.

### DISCUSSION

Physical confinement of the tumour cells and their products by cultivation in the restricted space of the capillaries enabled the cells to grow and proliferate in almost " pure " culture. Density of cell population appeared to be a decisive factor for the fate of the cultured tumour cells. On the one hand, a dense population caused cell degeneration and necrosis, presumably by the production of waste products and deprivation of essential metabolites. On the other, the tumour cells were mutually protective and beneficial. Cells too widely separated within a capillary, at distances of 0·5 to 1 mm., rarely survived more than 24 hours.

Prolongation of cell division required a considerably higher population density than did maintenance in good health. The correlations between density of tumour

---

EXPLANATION OF PLATE.

Photomicrographs of ascites tumour cells cultivated in glass capillaries.

FIG. 1.—Sarcoma 37. Cells cultured for 24 hours.

FIG. 2.—Sarcoma 37. Cells cultured for 48 hours.

FIG. 3.—Ehrlich carcinoma. Cells cultured for 72 hours.

FIG. 4.—Sarcoma 37. Cells cultured for 120 hours.

1 2

3 4

Powell.

cell population and survival and mitosis, respectively, established in these capillary cultures agreed with those found in open plasma cultures of the same strains of tumour cells (Powell, 1957*a*, 1957*b*). There was, however, a significant difference in the vitality of the cells cultured by the two methods. Both the duration of survival and cell division were much increased in capillary cultures in comparison with open cultures of similar densities of population. It was noticeable that the tumour cells in capillaries tolerated much greater separation from each other than in the coverslip cultures.

The hypothesis that the viability of cultured ascites tumour cells was largely dependent on the concentration of protective factors produced by the cells themselves was strengthened by these results. An optimum population density enabled the cells to produce local concentrations of these essential factors adequate for their needs. Capillaries of the dimensions specified allowed the cells to obtain fresh supplies of metabolites without dissipating too much of the diffusible substances and without causing a harmful concentration of waste products.

The absence of cell migration and rarity of transformation except in the immediate vicinity of the open ends of the capillaries may be significant in view of the physical confinement of the cultured cells. There would be small likelihood of a sharp diffusion gradient of cell products inside capillaries containing uniformly distributed cells.

It was concluded that ascites tumour cells cultured *in vitro* were themselves capable of forming the essential protective substances upon which their vitality depended. Evidence was also obtained that macrophages liberated similar or identical substances in apparently greater amounts than equal numbers of tumour cells.

### SUMMARY

A method for the cultivation of Ehrlich carcinoma and Sarcoma 37 ascites tumour cells in glass capillaries is described.

The enclosed cells survive and divide for significantly longer periods than when cultivated in open coverslip cultures at comparable densities of population. The cells in capillaries also tolerate significantly greater separation from each other.

These differences in vitality are attributed to confinement of the cells in the capillaries. This restricts the loss of soluble protective factors from the immediate environment of the cells.

I am much indebted to Mr. G. A. Butcher for his assistance with this work.

The expenses of these researches were defrayed from a block grant by the British Empire Cancer Campaign.

### REFERENCES

POWELL, A. K.—(1957*a*) *Brit. J. Cancer*, **11**, 274.—(1957*b*) *Ibid.*, **11**, 280.

SANFORD, K. K., EARLE, W. R. AND LIKELY, G. D.—(1948) J. nat. Cancer Inst., **9**,